# Dementia, Travel, and Tourism: A Scoping Review

**DOI:** 10.1177/14713012251363867

**Published:** 2025-08-13

**Authors:** Clarissa Giebel, Catherine V. Talbot, Marcus Hansen

**Affiliations:** 1 Department of Primary Care and Mental Health, 4591 University of Liverpool, Liverpool, UK; 2 NIHR Applied Research Collaboration North West Coast, Liverpool, UK; 3 Department of Psychology, Bournemouth University, Poole, UK; 4 Liverpool Business School, 4589 Liverpool John Moores University, Liverpool, UK

**Keywords:** dementia, tourism, travel, visitor economy

## Abstract

Background: Little is known about travelling and tourism for people living with or caring for someone with dementia. The aim of this scoping review was to synthesise the existing evidence on dementia, travel and tourism, the experiences of people with dementia and their carers, and how venues and businesses are dementia friendly. Methods: The review protocol was prospectively registered on PROSPERO [ID: CRD42023397637]. Four databases were searched for relevant literature in February 2024. Studies were included if they were published in English, Danish, or German, without any restrictions on publication date. Titles and abstracts, and full texts, were reviewed by two different research team members, and any disagreements were resolved in discussion with the wider team. Results: From 1,523 screened records, 13 papers were included. Evidence showed wide-ranging barriers for people with dementia and their carers to travel, often limiting the travel to local well-known places with adequate facilities or decisions on whether to travel solo as a carer. Seven studies focused on attitudes of businesses and tourist attractions and their implementations of dementia-friendliness. There was a notable lack of knowledge about dementia, and whilst most sites were willing to adapt existing attractions to include people with dementia, a lack of financial resources was the main barrier consistently referred to. Conclusion: Little remains known about the experiences of people living with dementia and their carers surrounding tourism and local, national, and international travel, and how different tourism organisations provide dementia-friendly support. With a focus on supporting people with dementia to remain as independent as possible, research needs to explore the population’s experiences, and how tourism destinations and modes of travel and transport could improve to be dementia-friendly and inclusive.

## Introduction

Dementia affects over 55 million people worldwide, with millions of family members and friends providing unpaid care (ADI, 2022). Dementia is an umbrella term for various different subtypes, the most common one being Alzheimer’s disease, and rarer forms including Lewy Body, Parkinson’s disease, vascular, and behavioural-variant fronto-temporal dementia, among others. Different dementia subtypes can be distinguished from one another by their symptomatology, with different levels of memory, processing speed, executive functioning, and other cognitive processes; initiative and performance of basic and instrumental activities of daily living (washing and feeding; preparing a meal and managing medication and finances, for example); motor functioning; and neuropsychiatric behaviours affected (such as hallucinations, agitation, and sleep disturbances) (Giebel et al., 2017; Gnanalingham et al., 1997; Irish et al., 2011). Thus, people living with dementia experience different needs based on their subtype, but also based on their personal needs, whilst travel and tourism still remains an important part of their independence.

When assessing independence in dementia, using transport is one of the key eight original instrumental activities of daily living (IADLs), as defined by Lawton and Brody (1969). Specifically, the level of independence ranges from travelling fully independently (car, public transport), to arranging a taxi, travelling on public transport accompanied by someone else, to lacking the ability or motivation to travel completely. Previous research focusing on Lawton and Brody’s gold-standard assessment of everyday functioning in dementia practice has shown that deteriorations in using transport (among three other IADLs) can be used as a predictor of one-year incident dementia risk (Barberger-Gateau et al., 1993). Moreover, Peres et al. (2008) showed that difficulties in using transport, as measured on the same scale, was significantly associated with incident risk of dementia two and five years later. From a dementia risk point of view, these findings clearly indicate the link between dementia and this daily or lifestyle activity.

From a post-diagnosis point of view, a small but recently growing evidence base has started to emerge on the experiences of dementia in the context of tourism (i.e. Connell & Page, 2019; Tomej et al., 2023; Wen et al., 2024). Tourism is increasingly seen as a potential remedy to address critical problems within society, such as wellbeing, quality of life and social isolation (McCabe & Qiao, 2020). Whilst tourism has already been recognised as important for people with dementia (Wen et al., 2024), tourism experiences remain littered with access constraints (Connell & Page, 2019a; Peterson et al., 2022). Dementia tourism is based on the values of accessible tourism, namely independence, equity and dignity and adheres to the social model of disability, in which it is society and the built environment that is considered disabling (Darcy & Dickson, 2009; McKercher & Darcy, 2018). Whilst dementia is not recognised as a disability, as a health condition it may contribute toward a sense of disability, similar to other neurological conditions such as autism (Connell & Page, 2019b). Considering the readiness of destinations to provide dementia-friendly tourism, Connell & Page (2019a, 2019b) reported online information about dementia friendly tourism and found limited information for UK destinations, highlighting the need for increased communication and readiness of tourism and tourist sites for people living with dementia. However, research seems to use diverse foci of travel and tourism, and to date, no review has synthesised the evidence base.

Many argue that tourism destinations are missing out on a competitive advantage by ignoring a growing demographic (Connell & Page, 2019; Vila et al., 2015; Wen et al., 2024). Specifically in the case of dementia, this consumer group is estimated to represent around 4% of the UK population, a figure that is expected to rise alongside a rapidly ageing population (Connell & Page, 2019b). It is also worth recognising that tourism destinations exist within local communities, meaning the benefits of tourism development in turn extend to local residents as well (Hartwell et al., 2018; Page & Connell, 2024). Importantly, access to leisure, such as tourism, is considered a social justice issue (Duignan et al., 2023) and a human right by the United Nations under the Universal Declaration of Human Rights of 1948 (McCabe & Diekmann, 2015; United Nations, 2015). The idea of dementia inclusivity is based on the UN Convention on the Rights of People with Disabilities, in which it is acknowledged that full participation in society is realised by the identification and subsequent removing of barriers (Page & Connell, 2024). The right to access is also protected under disability legislation in most countries, such as the UK’s Equality Act of 2010 (Randle & Dolnicar, 2019). Unfortunately, such legislation tends to be outdated and has limited compliance thresholds (Cloquet et al., 2018). In turn, people with dementia remain largely alienated from tourism and the associated benefits to quality of life and wellbeing.

The aim of this novel scoping review is to synthesise the existing evidence into dementia, travel and tourism, the experiences of people with dementia and their carers, and how venues and businesses are dementia friendly. Local, regional, national, and international travel and tourism represents a key aspect of daily life, and international travel in particular has become increasingly accessible to everyone, whilst advanced cognitive deterioration and behavioural symptomatology may affect people with dementia’s ability to do so. Thus, to ensure that people living with dementia can continue to travel and thus remain as independent as possible, it is important to understand the existing evidence and provide recommendations for travel and tourism in dementia.

## Methods

The protocol of this qualitative scoping review was registered on PROSPERO [ID: CRD42023397637]. A narrative approach was taken due to the review’s focus on lived experiences of travelling and tourism with dementia and dementia-friendly attitudes and adaptations of businesses and visitor attractions.

### Inclusion and Exclusion Criteria

Qualitative, quantitative, and mixed-method studies were included in this review. Studies were included if they focused on: people living with dementia or unpaid carers of people living with dementia; focused on travel and tourism experiences. We excluded studies of people without a diagnosis of dementia; unpaid carers caring for someone without dementia; not focusing on travel experiences (by train, car, taxi, tram, bus, plane, ferry). Studies which focused on driving were excluded from this review because the evidence base surrounding driving is very specific to a person’s cognitive functioning and the impact on their ability to drive (daily activity). No limits were placed on the type or stage of dementia.

### Search Strategy

We searched the following databases in February 2024: PubMed, PsycINFO, Scopus, Web of Science. Restrictions were applied to specify studies written in English or German language. No restrictions were placed on time of publication. The search terms were piloted before being used and developed in consultation with an experienced librarian: [dementia] AND ([tourism] OR [travel])**.** All records from searches were retrieved in Endnote and uploaded to Covidence, a web-based screening and data extraction tool, where duplicates were removed.

### Study Selection

Using Covidence, the titles and abstracts of all papers were screened by two reviewers against the inclusion criteria (CG, CT). Any discrepancies about included papers were discussed between the reviewers until consensus was achieved. Following Stage 1 screening, each full paper was read by two reviewers (CT, MH). As in Stage 1, any discrepancies were discussed until consensus was reached.

### Data Extraction

One researcher (CT) extracted the following data: population, sample size, country, year, travel type, travel length and destination, travelling alone or together, qualitative themes generated from each study.

### Data Synthesis

Narrative synthesis was applied to synthesise the findings of included studies. First, textual summaries of the findings from each study were produced by two reviewers (CG, CT). These summaries were then organised into distinct categories and sub-categories, to identify common themes and variations across included studies. Narrative descriptions were then produced for each category/sub-category.

## Results

### Overview of Included Studies

**Figure 1.**
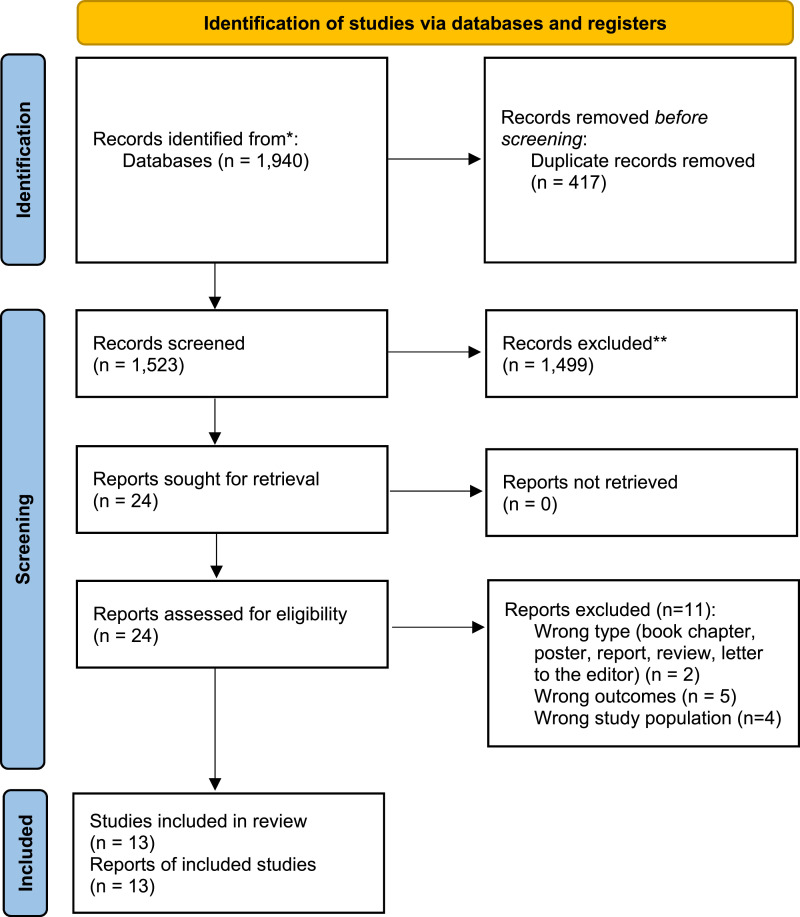
PRISMA Flowchart

From 1,523 records screened and 24 full-text papers read for inclusion/exclusion, 13 studies (Ashgar et al., 2020; Connell et al., 2017; Connell & Page, 2019a, 2019b; Gazzola et al., 2018; Innes et al., 2026; Johnston & Terp, 2015; Page et al., 2015; Peterson et al., 2022; Stewart et al., 2022; Tomej et al., 2023; Timmermans et al., 2020) were included in the final review (see PRISMA Flowchart in Figure 1). Eight of the included studies were conducted in the UK, with the remaining evidence gathered in Belgium, Italy, and the USA. The location of one study (Ashgar et al., 2020) was unclear. Of the included studies, seven studies focused on the experiences and attitudes of people living with dementia and unpaid carers surrounding travel and tourism (Ashgar et al., 2020; Gazzola et al., 2018; Innes et al., 2026; Johnston & Terp, 2015; Petersson et al., 2022; Timmermans et al., 2020; Tomej et al., 2023), whilst seven studies focused on Dementia-Friendly Tourism (DFT) of businesses and attractions/events (Page et al., 2015; Connell et al., 2017; Connell & Page, 2019a, 2019b; Stewart et al., 2022; Timmermans et al., 2020). Timmermans et al. (2020) focused on both aspects. Table 1 provides an overview of study characteristics for each included study.

**Table 1.** List of Included Studies and Key Outcomes

Authors	Country	Sample	Aim	Results
Asghar et al. (2020)	Unclear (UK or China)	327 people with dementia	Investigate the impacts of assistive technology (AT) assisted travel and tourism for people with dementia	ATs supported travel and tourism activities, resulting in a sense of improved achievements, independence, and safety
Connell and Page (2019a)	UK	1508 businesses in 135 dementia action alliances (DAAs) in coastal areas	Examine the activities of local businesses in the development of a dementia-friendly visitor economy	Challenges: Limited awareness of dementia, time to become dementia-friendly and train staff, turnover of staff, and funding to implement changes
				Action plans focused on training, running events or providing activities for people with dementia
				Adaptations: Raising awareness, auditing premises or processes, customer cards for bus travel, coin charts at cash trills, better menu design, and improved signage
Connell & Page (2019b)	UK	127 destination management organisation (DMO) websites and 32 DMO managers	Audit the accessibility provision of visitor destinations and understand knowledge and attitudes of DMO mangers towards developing dementia-ready organisations	63% knew the term ‘dementia-friendly’. Less than 50% of DMOs working on dementia-friendly initiatives. 70% saw the benefits of becoming dementia-friendly and two thirds felt it would make them more competitive
				Initiatives being developed: Beacon attractions and public sector transport operators, dementia friends training, accessible accommodation, access tourism groups, voluntary actions
				Barriers to becoming dementia-friendly: Uncertainty about member support, cost, and lack of information
Connell et al. (2017)	UK	31 businesses and 20 industry leaders and key service providers	Evaluate the awareness, perception, and experiences of businesses in developing a dementia-friendly visitor economy	1) Stage 1: Ignoring the problem
				2) Stage 2: Some awareness
				3) Stage 3: Building infrastructure
				4) Stage 4: Advocacy
				5) Stage 5: Key proposition
				6) Stage 6: Normalisation
Gazzola et al. (2018)	Italy	15 unpaid careers living in the North of Italy	Exploring experiences of people with dementia and unpaid carers	1) Characteristics of the person with dementia
				2) Caregivers characteristics
				3) Person with dementia and caregivers ‘needs
				4) Family relationship with leisure time
Innes et al. (2026)	UK	16 people with dementia; 19 unpaid carers; 13 older adults (without dementia)	Exploring experiences of people with dementia and unpaid carers	1) Personal
				2) Interpersonal
				3) Structural
Johnston and Terp (2015)	USA	5 couple dyads (person with dementia and partner)	Explore the dynamics in couples facing early-stage Alzheimer’s disease	Travel was a coping strategy that maintained ‘couplehood’ and affirmed their relationship
Page et al. (2015)	UK	20 tourism providers	Identify the feasibility, barriers and policy measures in developing dementia-friendly tourism	1) Awareness and understanding surrounding dementia
				2) Costs
				3) Extra effort
				4) Ongoing development
Page et al. (2023)	UK	40 business interviews, 11 business observations and site audits	Understand how organisations operationalise concepts and connect with visitor wellbeing through their dementia friendly ambitions and activities	Site audits
				Most sites had accessible websites displaying pre-trip information and accessibility issues
				Dementia training was not publicly displayed due to incomplete staff training
				Several sites offered accessibility information, services, and visual guides
				Business interviews
				1) Nature and the outdoors
				2) Transformative actions
				3) Transformative practice
				4) Barriers and challenges
Peterson et al. (2022)	USA	48 people with dementia; 176 travel companions	Explore experiences of people with dementia and caregiving travel companions using U.S airports	1) Airport security and staff interactions can help or hinder positive air travel experiences
				2) Problems with airport navigation
				3) Unaccommodating physical spaces
Stewart et al. (2022)	UK	8 employees across 7 arts and culture organisations	Explore how arts events tailored for people with dementia can help those with a diagnosis to live well	1) Sociopolitical impact
				2) Event planning and execution
				3) Community outreach and engagement
Tomej et al. (2023)	UK	21 Tripadvisor, 49 reddit, 20 dementia talking point, 11 ALZ connected threads on travel information needs of people with dementia and unpaid carers	Explore the travel information needs of people with dementia and unpaid carers	1) To travel or not to travel
				2) Choice of destination(s) and activities
				3) Mobility and transport
				4) Communication
				5) The (not so) mundane and connecting with everyday life
				6) Temporary carer replacement
Timmermans et al. (2020)	Belgium	2 people with dementia; 10 unpaid carers; 10 entrepreneurs	Exploring experiences of people with dementia and unpaid carers	Entrepreneurs:
				1) How society views people with dementia
				2) The importance of activities
				3) Providing an accessible and suitable offer
				4) Chances/opportunities in developing and providing dementia-friendly activities
				PLWD and their informal carers:
				1) The need for suitable leisure activities
				2) The preconditions for making activities suitable for PLWD
				3) Being a member of society
				4) Chances and opportunities in dementia-friendly leisure

### Experiences and Attitudes of Travel and Tourism for People Affected by Dementia

Seven studies investigated the experiences and attitudes of travel and tourism for people affected by dementia (Ashgar et al., 2020; Gazzola et al., 2018; Innes et al., 2026; Johnston & Terp, 2015; Petersson et al., 2022; Timmermans et al., 2020; Tomej et al., 2023). Informed by previous work on leisure participation (e.g., Innes et al., 2026), barriers and facilitators were grouped into three categories: (1) Intrapersonal factors (i.e., psychological states and personal preferences as well as personal relationships); (2) Interpersonal factors (i.e., interactions and relationships); (3) Structural factors (i.e., broader environmental and organisational aspects of travel and tourism).

#### Intrapersonal Factors

A common barrier to engaging in travel and tourism activities was anxiety about the person with dementia becoming lost. In Innes et al.’s (2026) focus group study, this worry dissuaded family members from taking people with dementia on vacations. For some carers this concern became a reality, with more than a third of participants in Peterson et al.’s (2022) study having experienced being separated at the airport, often occurring at security checks despite prior communication with Transportation Security Administration Agents.

Despite these concerns, across all seven studies participants expressed a willingness to engage in travel and tourism. While Peterson et al.’s survey (2022) showed that very few participants had ceased travelling altogether, the majority did report adjustments in their travel habits post-diagnosis. These adjustments included refraining from solo travel, reducing the frequency of trips, and favouring direct flight options. The motivation to engage in these activities, as noted by Gazzola et al. (2018) and Timmermans et al. (2020), was the opportunity for mutual enjoyment and potential respite. However, willingness to participate in tourism activities has been found to vary based on individual circumstances. Following qualitative interviews in the North of Italy, Gazzola et al. (2018) identified factors such as the characteristics of the person with dementia (e.g., behavioural problems, level of cognitive impairments), carers’ characteristics (e.g., perception of tourism as beneficial), the needs of the person with dementia and carer (e.g., need to enjoy time together), and their relationship with leisure time (e.g., past experiences of tourism) as shaping willingness to engage in such activities.

To overcome potential barriers in travelling, Ashgar et al. (2020) reported positive views and experiences of using assistive technology when travelling with dementia. This entailed easier communication and access to travel. However, the study falls short of notable key details including which country the 300+ people with dementia strong survey was conducted in. This is important as different countries may experience different levels of dementia-friendly tourism and travel. A lack of detail about the ethics committee providing ethical approval and how people with dementia were approached in detail further adds to the limited quality of this study and representativeness.

When questioned more broadly about what a diagnosis of dementia means for couples’ relationships, where one spouse was living with dementia, findings by Johnston and Terp (2015) advance these findings. Whilst not specifically asked about travel, this emerged as a key aspect that shaped relationships also beyond the dementia diagnosis. For three of six interviewed couples, the diagnosis made them take extensive trips across the world. For others, they were more spontaneous in deciding to travel more, showing that the diagnosis can have a significant, positive, impact on couples who are dealing with dementia. This is contrary to findings by Tomej et al. (2023). Utilising a different methodology as opposed to conducting qualitative interviews about people’s experiences, analysis of posted messages in travel and dementia fora further showed that this group experiences travel and holidays with dementia as ‘the last holiday’ wanting to engage with previous memories. Travelling was not considered something positive to continue to engage in, as transport to the destination was considered stressful, particularly airports. In some instances, carers decide to travel without the person with dementia, and chose respite but were often plagued by feelings of guilt.

#### Interpersonal Factors

Across four studies, interactions with the general public or members of staff were noted as challenges (Innes et al., 2026; Peterson et al., 2022; Timmermans et al., 2020; Tomej et al., 2023). Feelings of embarrassment were reported in two studies, resulting from individuals with dementia behaving in ways which may be misunderstood by others (Innes et al., 2026; Timmermans et al., 2020). Consequently, carers in Innes et al.’s (2026) study reported disclosing the dementia diagnosis to others as a pre-emptive measure in public situations as well as a desire for others to be more tolerant. Moreover, many participants in Peterson et al.'s (2022) study reported negative interactions with airport security staff, including experiences of being shouted at which increased stress levels. However, participants also encountered positive interactions with staff members who were polite, patient, and accommodating. In light of these challenges, participants in both Peterson et al. (2022) and Innes et al.’s (2026) studies called for increased staff training to promote greater awareness and understanding of dementia. Similarly, forum users in Tomej et al.’s (2023) ethnography study praised lanyards designed to communicate hidden disabilities to staff, though many remained uncertain about how widely recognised these tools actually were.

#### Structural Factors

Accessibility of transport was a key concern among people affected by dementia. For example, some people with dementia in Innes et al.’s (2026) study could no longer drive, leaving them reliant on limited bus services, which often involved lengthy journeys. Additionally, participants also reported issues with rail services, including physical access issues, navigational difficulties, and short timeframes between connecting trains; issues which were further exacerbated for those with mobility issues.

Another recurring issue across studies was the accessibility of restroom facilities, particularly the inadequacy of signage and lack of family restrooms. As reported by Peterson et al. (2022), a lack of family restrooms was burdensome for carers and heightened concerns about becoming separated. Furthermore, two studies reported that loud and overstimulating environments were overwhelming for people with dementia, highlighting the importance of quiet spaces in mitigating dementia-related behaviours associated with sensory overload (Peterson et al., 2022; Timmermans et al., 2020).

In light of the complexities associated with travel and tourism, people with dementia in Innes et al.’s (2026) study expressed a preference for local and dementia-friendly venues equipped with all amenities. Participants identified ways businesses in the visitor economy could improve provision, including organised coach trips, museum visits, vacations for dementia households, and dementia-friendly hotels. In Belgium, Timmermans et al. (2020) also noted the importance of supporting carers, emphasising the need for reliable care services within the visitor economy. This support was described as not only vital when carers wish to engage in activities with people with dementia but also when they require respite. Tomej et al. (2023) extend these findings, reporting that unpaid carers had outstanding travel information needs, and were thus restricted in engaging in travelling with their relative.

### Dementia-Friendly Tourism of Businesses

Seven studies explored the experiences and attitudes of tourism and travel destinations and businesses about their dementia friendliness using either quantitative or qualitative approach (Connell et al., 2017; Connell & Page, 2019, 2019b; Stewart et al., 2022; Timmermans et al., 2020).

Page et al. (2015) conducted a scoping study with tourism businesses in a coastal resort (Bournemouth) in the UK. After having derived interview questions with people with dementia and their carers, they interviewed 20 businesses operating in the visitor economy across the city in 2012. Page et al. (2015) evidenced different views on whether people with dementia should participate in the same activities as everyone else, or whether they need to be assisted specifically. Businesses expressed divergent attitudes towards dementia – those with personal experiences were more sympathetic and understanding and supportive; those without personal experiences but only professional experiences of disabilities tended to be more negative towards dementia. Some expressed fear of negative impacts on their businesses and other customers with people with dementia present, thus stigmatising the condition, and preferred people with dementia to only attend with a carer. These findings were corroborated by findings from Connell et al. (2017), having surveyed 31 businesses in the UK, all of which reported a lack of financial resources and high costs associated with adapting attractions and events for people with dementia. However, all were interested in being dementia-friendly, thus showing a more positive approach towards integration than evidence from Page et al. (2015).

Two subsequent studies collecting data from a broader sample of business across the country, not restricted to Bournemouth, further supported these findings. Connell and Page (2019a) evidenced a lack of awareness of dementia and high staff turnover impeding creating dementia-friendly tourism in over 1500 surveyed businesses across 135 Dementia Action Alliances in coastal areas. Additionally exploring the readiness of destination management operators about their dementia-friendliness, Connell & Page (2019b) showed predominantly an interest in becoming dementia-friendly (70% of organisations), whilst less than 50% were working on dementia-friendly initiatives. Costs and lack of resources appeared to be a recurring theme in studies focusing on dementia-friendly initiatives and tourism, with managers reporting this as a key barrier in becoming dementia friendly (Connell & Page, 2019b), similar to Page et al. (2015) and Connell et al. (2017), as well as Timmermans et al. (2020).

Investigating specifically how different arts and culture attractions are being dementia-friendly, reported different levels of support and information provision for people with dementia in historic and religious sites, nature outdoor events, and estates and gardens for visitors. This lack of dementia-friendly support was further evidenced by Stewart and colleagues (2022), who also reported a lack of dementia accessibility awareness in eight event organisers from seven different arts and culture organisations, including those organising music festivals. Corroborating previously reported attitudes towards making existing events dementia-friendly as opposed to creating separate events for people with dementia and their carers, interviewees highlighted how using a grass-roots approach and conducting outreach work with local communities appeared to be the most effective and inclusive way of achieving this goal (Stewart et al., 2022). Whilst the authors suggested a new model to capture these attitudes of dementia-friendliness in arts events, findings were only based on eight event organisers and the adapted model only included stakeholders as a new addition, which limits the value of this proposed new model.

## Discussion

This is the first scoping review to synthesise the limited but recently burgeoning evidence base on travel and tourism in dementia, specifically focusing on the experiences of people living with dementia and their unpaid carers and the attitudes of the tourism industry and local businesses towards being dementia friendly. Limited evidence was found for inclusion, with five qualitative studies reporting on the limited support experiences and available travel and tourism options for people with dementia and their carers, whilst more limited evidence highlighted the often lack of willingness of tourism venues to become dementia friendly.

Travelling with dementia and with a relative with the condition was often an experience fraught with difficulties. In the still limited but growing recent evidence, carers and people with dementia highlighted many considerations prior to planning a leisure outing and visiting tourism venues, and were often discouraged from travelling (Gazzola et al., 2018; Innes et al., 2026; Petersson et al., 2022; Timmermans et al., 2020). These included intrapersonal factors such as concerns about being separated when travelling; interpersonal factors including difficult engagement with the general public and a lack of understanding of dementia; and structural factors, such as inaccessible infrastructure, including transport, accommodation, attractions etc. That facilitate the needs of the person with dementia. These constraints are similar to those highlighted in the wider accessible tourism literature, in which negative attitudes toward people with disabilities and a lack of awareness of disability in general from tourism stakeholders, in particular, are considered the roof of all constraints (see Duignan et al., 2023; McKercher & Darcy. 2018).

Many unpaid carers had outstanding travel information needs, and were thus restricted in engaging in travelling with their relative (i.e. Tomej et al., 2023). In light of the limited evidence painting a mostly negative picture of tourism and travelling with dementia, Dementia-Friendly Communities (DFC) could offer one approach to improving accessibility and support for people with dementia and their carers when wanting to engage in these activities (Connell & Page, 2019a). Likewise, the notion that thriving destinations also benefit the community within which the destination sits [and vice versa] (Hartwell et al., 2018; Page & Connell, 2024) makes for a further compelling argument for the creation of dementia-friendly destinations.

Being unable to engage in previously enjoyed leisure activities, such as travelling to the seaside or going to museums or other tourism-based activities, are considered a key part of modern-day life. Engaging in leisure and tourism activities is linked to increased levels of well-being for the general population (McCabe & Qiao, 2020; Wen et al., 2024), with limited and poor quality qualitative evidence for dementia to date. Whilst dementia affects the ability to initiate and perform daily activities, such as managing finances and preparing a hot meal (Giebel et al., 2017), being supported to continue engaging in these daily activities is arguably equally important as engaging in hobbies and going on vacation or day trips. This is in line with the work of Wen et al., (2024), which recognises tourism as a non-pharmaceutical treatment of dementia. Thus, one way to support people with dementia and unpaid carers to continue engaging in tourism-based activities, is by linking this to social prescribing, which shares several similarities with the concept of social tourism (McCabe & Qiao, 2020). Social tourism is defined as “all activities, relationships and phenomena in the field of tourism resulting from the inclusion of otherwise disadvantaged and excluded groups in participation in tourism” (Minnaert et al., 2012). In turn, social tourism stimulates social and economic development, whilst simultaneously delivering critical health and wellbeing benefits to disadvantaged and otherwise excluded tourists (Cisneros-Martinez et al., 2018; McCabe & Qiao, 2020).

There is a burgeoning evidence base on social prescribing and positive findings on mental health outcomes for the general population (Cooper et al., 2022), whilst a recent synthesis of social prescribing initiatives for older adults found a lack of structure and framework to provide clear evaluations of their effectiveness (Hamilton-West et al., 2020). However, Hamilton-West et al. (2020) conclude that social prescribing has the potential to act as a suitable social strategy employed within the NHS to support older adults’ health and well-being. One recently evidenced social prescribing service for people with dementia and unpaid carers specifically focused on providing mental and physical health support and activities in an area of the North West of England (Giebel et al., 2021). Following up participants over three and six months, compared to baseline, well-being was significantly higher at both follow-ups, whilst lack of transport was considered a barrier to continuation of the service. Whilst transport in this case was not used for a tourism-based social prescribing activity, findings illustrate the need for adequate transport infrastructure for people with dementia and their carers to partake in leisure activities. More research needs to be done to investigate the link between social prescribing and tourism for dementia, and its effect on health and well-being.

Whilst we utilised a broad search strategy for this scoping review, only 13 papers were eligible for inclusion, highlighting the dearth of evidence available to date. This limited evidence base was also generally of poor quality, often lacking clarity of methods and rigorous data analysis. Existing evidence was not representative of the population of people living with dementia and their carers, and was focused on specific cities as opposed to national recruitment of participants. In addition, evidence was lacking on air and international travel, without a distinction in the existing literature on day trips, short breaks, and longer vacations. These limitations highlight an urgent need for robust dementia research focusing on lived experiences of travel and tourism.

## Conclusions

Based on the limited evidence base to date, findings to date suggest that people with dementia and their carers are not supported adequately to enjoy touristic leisure activities and travel to get there. This was echoed in the experiences and attitudes of small tourism businesses, with a high stigma surrounding dementia still prevailing. Given the limited representativeness and quality of existing research, future research needs to explore the experiences of people living with dementia and their carers across the UK, focusing on different types of travel (including regional, national, and international), as well as different types of tourism activities (including day trips, short breaks and longer vacations – nationally or abroad), and explore any support needs. Continuing activities that were enjoyed prior to the dementia diagnosis are vital to maintaining a good quality of life, and should thus be actively facilitated by the tourism industry and could be linked to the NHS-based social prescribing system.
